# Brain Morphological and Functional Changes in Adenomyosis with Pain: A Resting State Functional Magnetic Resonance Imaging Study

**DOI:** 10.3390/jcm11185286

**Published:** 2022-09-07

**Authors:** Xue Jiao, Ming Yuan, Qiuju Li, Yufei Huang, Miaomiao Ji, Jing Li, Shumin Yan, Hao Sun, Xinyu Wang, Zangyu Pan, Qianhui Ren, Dawei Wang, Guoyun Wang

**Affiliations:** 1 Department of Obstetrics and Gynecology, Qilu Hospital of Shandong University, Jinan 250012, China; 2 Department of Gynecology, Shandong Provincial Hospital, Jinan 250021, China; 3 Medical Integration and Practice Center, Shandong University, Jinan 250012, China; 4 Maternal and Child Health Care Hospital of Shandong Province, Jinan 250014, China; 5 Department of Radiology, Qilu Hospital of Shandong University, Jinan 250012, China; 6 Department of Epidemiology and Health Statistics, School of Public Health, Shandong University, Jinan 250012, China; 7 Institute of Brain and Brain-Inspired Science, Shandong University, Jinan 250012, China

**Keywords:** adenomyosis, brain morphology, brain function, pain, resting-state fMRI

## Abstract

The absence of clinically objective methods to evaluate adenomyosis-associated pain and the poor understanding of its pathophysiology lead to treatment limitations. We conducted a resting-state functional magnetic resonance imaging study with 49 patients with pain-related adenomyosis and 30 pain-free controls to investigate brain morphological alterations and regional dysfunctions in patients with pain-related adenomyosis. These patients had significantly higher scores for anxiety and depression than the control group (*p* < 0.05). They also had a lower gray matter volume (GMV) in the bilateral insula, left angular gyrus, precuneus, left inferior temporal gyrus, and left postcentral gyrus (*p* < 0.05, AlphaSim corrected). Similarly, decreased voxel-mirrored homotopic connectivity was observed in the bilateral insula, posterior cingulate cortex, middle frontal gyrus, and postcentral gyrus in the adenomyosis patient group (*p* < 0.05, AlphaSim corrected). Regional homogeneity showed significant differences mainly in the bilateral cerebellum, left inferior frontal gyrus, medial prefrontal cortex, and posterior cingulate gyrus. Correlation analysis showed that the degree of depression in patients with adenomyosis was negatively correlated with the GMV of the left angular gyrus. The results show that these patients exhibited changes in multiple brain regions associated with pain as well as emotion perception and processing.

## 1. Introduction

Adenomyosis (AM), characterized by the invasion of endometrial mucosa within the myometrium, is a common, benign, and chronic gynecological disorder with an indeterminate etiology [1,2]. AM symptoms include abnormal bleeding, pelvic pain, and infertility [3]. Pelvic pain, as one of the key features of AM, including dysmenorrhea, non-clinical pelvic pain, dyschezia, and dyspareunia, significantly negatively impacts on women’s quality of life during their reproductive years and even causes a tremendous burden on the social economy [4]. However, the pain experienced varies greatly between individuals [5]. Furthermore, the pathophysiology of AM-associated pain is poorly understood [2].

Interestingly, a strong association was found between AM and endometriosis (DIE), and there were clinical and pathologic similarities and distinctions between the two conditions, especially dysmenorrhea and chronic pelvic pain [6]. Our previous studies suggested that the density of nerve fibers, including sensory Aδ, autonomic, and sensory C fibers, increased at the ectopic lesion in the endometriosis model of rats, reducing the pain thresholds in patients and animals with endometriosis [7,8]. Moreover, the pain mediators sensitize sensory neurons and trigger a pain-signal cascade [9]. Information is conveyed to the central nervous system via the dorsal root ganglia, through the spinothalamic tract, spinoparabrachial amygdala projections, and spinoreticular pathways, resulting in persistent alterations in the brain morphology and function [10]. AM-associated pain might be associated with peripheral and central sensitizations [11]. In addition, the patients with AM had a higher risk of anxiety and depression with a worse response to pain treatment than those without pain [12,13]. This suggests that central sensitization and emotional processing play an important role in AM, and that the vicious circle leads to chronic pain.

Resting-state functional magnetic resonance imaging (fMRI) is based on blood-oxygenation-level-dependent (BOLD) spontaneous activity [14]. Analytical methods, such as regional homogeneity (ReHo) [15] and voxel-mirrored homotopic connectivity (VMHC), are widely used to reflect regional function in patients with chronic pain [16,17,18,19]. Voxel-based morphometry (VBM), a method to quantify the gray matter volume (GMV) difference [20] in several disorders, such as osteoarthritis, reflects the brain morphological changes [21]. For example, patients with osteoarthritis with pain showed lower GMV in the anterior cingulate and primary motor cortex with the method of VBM, and back pain with lumbar disk herniation was found to have global brain function disrupted using resting state fMRI [17,21]. However, it is reported that the incidence of dysmenorrhea in patients with AM was between 50% and 93.4% and seriously affected the patient’s quality of life. Few studies have focused on the brain abnormalities in patients with AM.

This study aims to investigate brain morphological and functional changes in AM patients with pain, and the correlation between the changes in the brain and the degree of anxiety and depression. Ultimately, we aimed to investigate the neural mechanism of pain in patients with AM and provide a more objective pattern of pain assessment for AM.

## 2. Materials and Methods

This study was registered on chictr.org.cn (ChiCTR2100045373, accessed on 13 April 2021) and approved by the Medical Ethics Committee of the Qilu Hospital of Shandong University; all of the participants signed informed consent forms. The participants were enrolled in the obstetrics and gynecology ward of the Qilu Hospital of Shandong University between May 2020 and April 2021.

### 2.1. Participants

This study included 50 right-handed patients with AM. The inclusion criteria for AM patients were as follows: (1) age between 20 and 50 years; (2) gynecological ultrasound findings suggesting AM according to the Morphological Uterus Sonographic Assessment (MUSA); (3) numeric pain rating scale score ≥4 and dysmenorrhea or chronic pelvic pain of at least 6 months duration; and (4) requiring surgery. In addition, the AM diagnosis confirmed only by postoperative pathology and no ovarian endometriosis cysts or deep endometriosis were found during surgical exploration. The exclusion criteria were as follows: (1) pregnancy or lactation; (2) presence of other chronic pain conditions [22], including gynecological factors such as chronic pelvic inflammatory disease, adnexal mass, leiomyoma, and pelvic congestion syndrome, as well as surgical (chronic appendicitis, adhesions), urological (interstitial cystitis, chronic urinary inflammation, urolithiasis, urethra syndrome), and gastrointestinal (irritable bowel syndrome, constipation, inflammatory bowel diseases) factors; (3) presence of neurological or psychiatric conditions; (4) use of oral contraceptives, hormonal supplements, traditional Chinese medication, or the intake of central-acting medications during the last 3 months before fMRI; and (5) presence of fMRI contraindications (e.g., claustrophobia). Thirty right-handed controls with cervical intraepithelial neoplasia requiring cervical conization were recruited during the study period, and gynecological ultrasound was performed to exclude AM and other uterine diseases. The controls had no neurological or mental illnesses and were similar to the patients with AM in terms of age, region, and social background.

All of the participants were evaluated by a gynecologist and underwent conventional MRI scans to exclude anatomical brain abnormalities. Demographic data and questionnaires were collected, and fMRI was performed pre-operatively. One patient with AM was excluded because of an image acquisition error. The final imaging and correlation analyses included 49 patients with AM and 30 controls.

### 2.2. Clinical Variable Measures

Before the study, all of the participants were informed of the study methods, procedures, objectives, and potential risks. Demographic information, including age, body mass index (BMI), menstrual status, and childbearing history, was obtained through an interview and clinical examination by a professional gynecologist. Additionally, the clinical data, such as hemoglobin (HGB) level and uterine volume, were collected before the surgery.

The numeric rating scale (NRS), with scores ranging from 0 to 10, was used to assess the mean pain intensity in the past 6 months, including dysmenorrhea, dyspareunia, dyschezia, and non-menstrual pelvic pain [23,24]. All of the participants completed the standardized measures for anxiety and depression. The detailed questionnaires included the Self-Rating Anxiety Scale (SAS) [25] and the Self-Rating Depression Scale (SDS) [26]. All of the questionnaires were used in validated Chinese versions.

### 2.3. Imaging Data Acquisition and Processing

MR images were acquired using a Siemens Verio 3.0 Tesla MR scanner (Erlangen, Germany). To minimize head movement, tight and comfortable foam padding was used, and earplugs were also provided to all of the participants to muffle the scanner noise. Resting-state fMRI data were obtained using a gradient-echo single-shot echo planar imaging sequence with the determined imaging parameters (File S1, Supplementary Materials). During the fMRI scanning, all of the participants were instructed to keep their eyes closed, to stay as still as possible, think of nothing in particular, and not fall asleep. Sagittal three-dimensional T1-weighted images were acquired using a magnetization-prepared rapid acquisition gradient echo sequence (File S1).

The fMRI data were preprocessed using Statistical Parametric Mapping (SPM8, http://www.fil.ion.ucl.ac.uk/spm (accessed on 1 August 2019) and Data Processing and Analysis (DPABI) for (Resting-State) Brain Imaging [27] (File S1). The standardized ReHo map of each participant was generated by calculating the Kendall coefficient concordance of the time series of a given voxel with those of its nearest neighbors (27 voxels) in a voxel-wise manner. Subsequently, spatial smoothing with a 6-mm full width at half maximum isotropic Gaussian kernel was performed to reduce the noise in the normalized functional images. The filtered time series of a given voxel was converted to a frequency domain using a fast Fourier transform process. The details of VMHC computation were expounded in a previous study [28]. Individual VMHC maps were generated for each participant by computing the Pearson correlation between a given voxel and a corresponding voxel in the contralateral hemisphere. For subsequent statistical analysis, Fisher’s r-to-z transformation was applied to improve the normality of the correlation.

### 2.4. Statistical Analyses

The Statistical Package for the Social Sciences (version 21.0, IBM Corp., Armonk, NY, USA) was used for the statistical analyses, and the level of significance was set at *p* < 0.05. An independent-samples *t*-test was used to compare the demographic characteristics and the clinical data of the two groups for continuous variables following a normal distribution, and the Mann–Whitney U test was used for the nonparametric continuous variables. Moreover, the chi-square test was used to compare the categorical variables. The significant differences in the fMRI statistical results were set at *p* < 0.05 (with a combined threshold of *p* < 0.05 and a minimum cluster size of 46 voxels), which were corrected using the AlphaSim program in REST plus software.

Correlation analyses were applied to detect the associations between the clinical characteristics (e.g., pain intensity and SAS and SDS scores) and mean GMV and altered function in all of the participants within each region of interest (*p* < 0.05, Bonferroni correction). Pearson’s correlation was applied to normally distributed data.

## 3. Results

### 3.1. Demographic and Clinical Characteristics

The demographic information and clinical characteristics of all of the participants are listed in Table 1. All of the participants were menstruating and had regular periods; however, there was no significant difference between the two groups (29.88 ± 8.85 vs. 28.80 ± 4.06, *p* > 0.05). There was no significant difference between the AM and control groups in terms of age, BMI, menstrual status, and childbearing history (*p* > 0.05). In contrast, the proportion of menorrhagia in the AM group was higher than in the control group (*p* = 0.001). In addition, the AM group had lower HGB levels and greater uterine volume than those of the control group (*p* < 0.001).

The pain intensity of the AM group was higher than that of the pain-free group (8.40 ± 1.78 vs. 0.43 ± 0.82, *p* < 0.001). As expected, there were significantly higher SAS and SDS scores in the AM group than in the control group (*p* < 0.05).

### 3.2. Brain Structural Differences between Patients with AM and Pain-Free Controls

**Figure 1:**
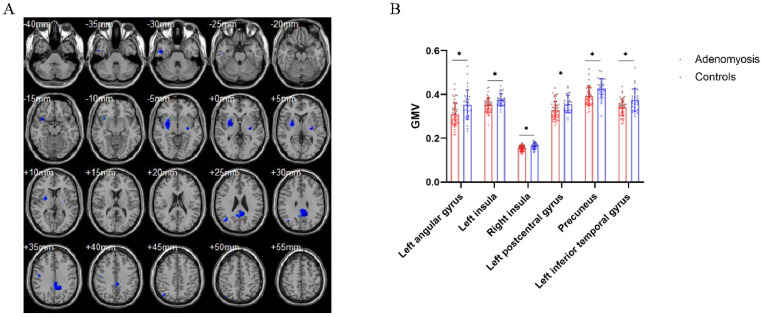
Brain structural differences between patients with adenomyosis and pain-free controls. (**A**) Brain regions with decreased gray matter volume (GMV) in patients with adenomyosis compared to pain-free controls, including the left angular gyrus, precuneus, left inferior temporal gyrus, left postcentral gyrus and bilateral insula. (**B**) Statistic diagram of different GMV of brain regions between patients and controls. * *p* < 0.05, AlphaSim corrected.

**Figure 2:**
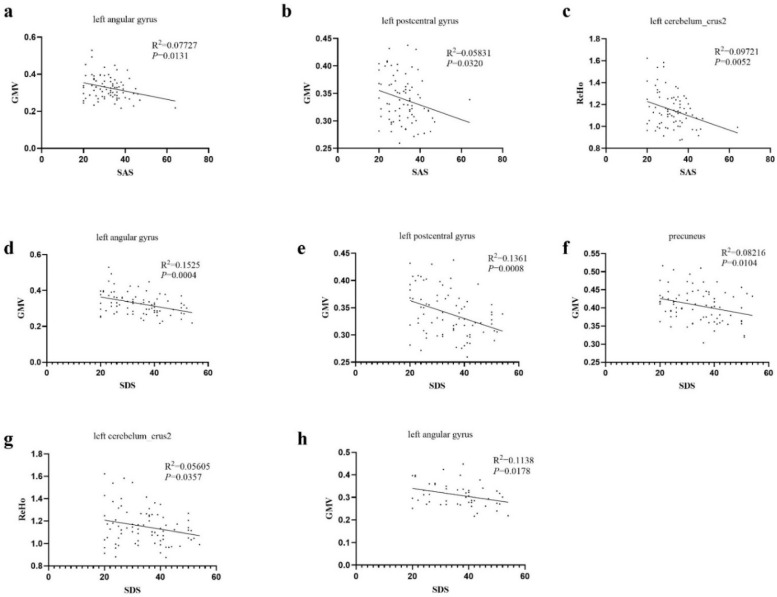
Correlations between degree of anxiety, depression, and abnormality of brain structure and function. (**a**) In all participants, the degree of anxiety was negatively correlated with the GMV of the left angular gyrus. (**b**) In all participants, the degree of anxiety was negatively correlated with the GMV of the left postcentral gyrus. (**c**) In all participants, the degree of anxiety was negatively correlated with the regional homogeneity (ReHo) of the left cerebellum. (**d**) In all participants, the degree of depression was negatively correlated with the GMV of the left angular gyrus. (**e**) In all participants, the degree of depression was negatively correlated with the GMV of the left postcentral gyrus. (**f**) In all participants, the degree of depression was negatively correlated with the GMV of the precuneus. (**g**) In all participants, the degree of depression was negatively correlated with the ReHo of the left cerebellum. (**h**) In patients with adenomyosis, the degree of depression had a significant negative correlation with the GMV of the left angular gyrus.

The patients with AM had significantly lower GMV in the bilateral insula and sensory and motor cortices, such as the left angular gyrus, precuneus, and left inferior temporal gyrus, along with the left postcentral gyrus (Figure 1, *p* < 0.05, AlphaSim corrected) than that of the control group. No brain regions showed greater GMV in patients with AM than in pain-free participants. Furthermore, both the SAS and SDS scores were negatively correlated with the GMV of the left angular gyrus and left postcentral gyrus (Figure 2a–c,e). The SDS score was also negatively correlated with the precuneus GMV (Figure 2f). More importantly, the analysis showed that the SDS scores in patients with AM had a significant negative correlation with the GMV of the left angular gyrus (Figure 2h).

### 3.3. Aberrant Interhemispheric Functional Connectivity between AM Patients and Pain-Free Controls

**Figure 3:**
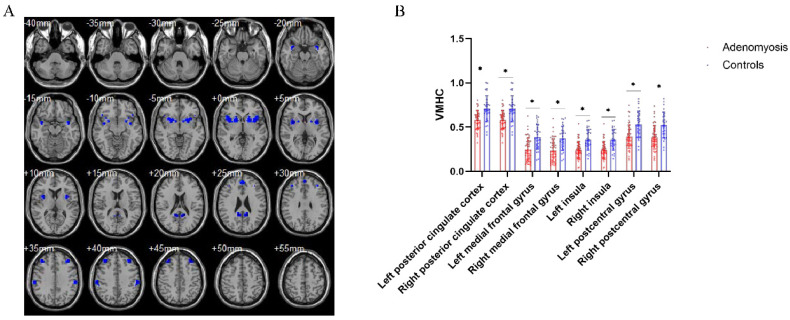
Aberrant interhemispheric functional connectivity between patients with adenomyosis and pain-free controls. (**A**) Brain regions with decreased voxel-mirrored homotopic connectivity in patients with adenomyosis compared to pain-free controls, including the bilateral posterior cingulate cortex, medial frontal gyrus, insula and postcentral gyrus. (**B**) Statistic diagram of different VMHC of brain regions between patients and controls. * *p* < 0.05, AlphaSim corrected.

The AM group exhibited decreased VMHC in the bilateral posterior cingulate cortex and medial frontal gyrus. Similar to the VBM results, decreased VMHC was observed in the bilateral insula and postcentral gyrus in the patient group (Figure 3). No brain regions showed increased VMHC in the patient group compared to the control group. Furthermore, correlation analysis was not statistically significant.

### 3.4. Brain Regional Homogeneity Differences between AM Patients and Pain-Free Controls

**Figure 4:**
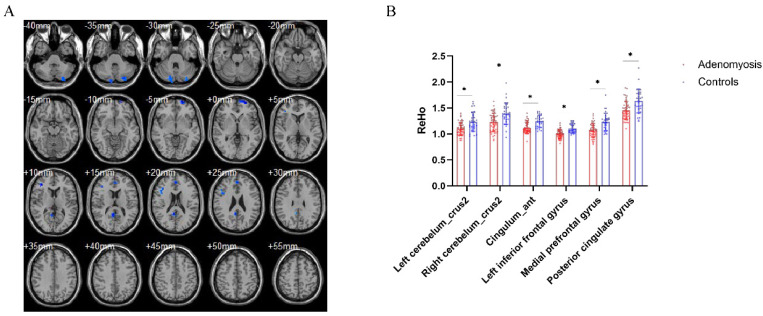
Differences in brain regional homogeneity between patients with adenomyosis and pain-free controls. (**A**) Brain regions with decreased ReHo in patients with adenomyosis compared to pain-free controls, including the bilateral cerebellum, left inferior frontal gyrus, medial prefrontal cortex, and posterior cingulate gyrus. (**B**) Statistic diagram of different ReHo of brain regions between patients and controls. * *p* < 0.05, AlphaSim corrected.

**Figure 5:**
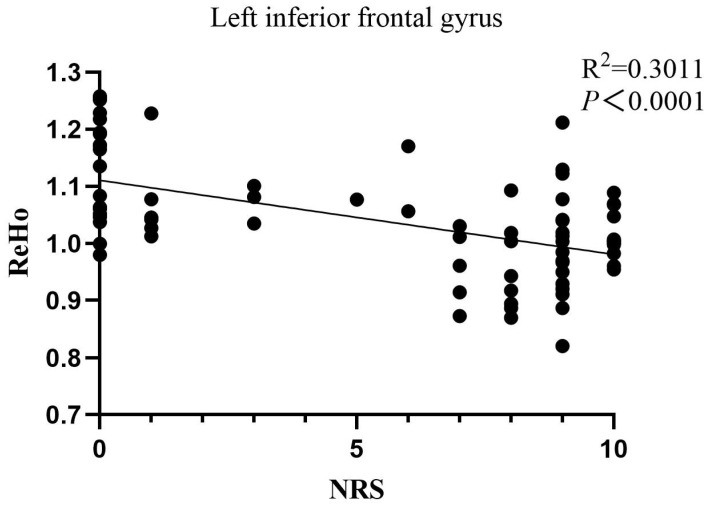
Correlation between the pain intensity and ReHo value of the left inferior frontal gyrus.

Brain ReHo differences between the groups were mainly located in the bilateral cerebellum, left inferior frontal gyrus, medial prefrontal cortex, and posterior cingulate gyrus relative to the controls (Figure 4). However, no higher ReHo values were significantly different between the two groups. In the correlation analysis, NRS was strongly negatively correlated with the left inferior frontal gyrus (R^2^ = 0.3011, *p* < 0.0001) (Figure 5). The SAS and SDS scores were negatively correlated with the left cerebellum (Figure 2c,g). There was no significant association between the aberrant structure and the dysfunction of brain regions and other clinical characteristics.

## 4. Discussion

Our study suggests that the patients with pain-related AM had a significantly lower GMV and decreased functional activities in several brain regions associated with pain perception and emotion regulation. The results were verified by different analyses, including VBM, VMHC, and ReHo. All of the participants’ degrees of pains correlated with several brain regions. Additionally, dysfunction in the left angular gyrus was significantly correlated with the SDS scores in the patients with pain-related AM.

The AM patients with dysmenorrhea and chronic pelvic pain are more sensitive to experimental pain outside of menstruation than the pain-free women, a phenomenon known as hyperalgesia [29,30]. Studies focusing on peripheral sensitization have demonstrated increased nerve-fiber density, neuropeptides, and neurotransmitter receptors in the lesions of patients with AM [8]. Central sensitization results in a decreased response threshold and dysfunction of neuronal activities [29]. It is hypothesized that the dysregulation of the brain regions or networks associated with pain regulation plays a predominant role in central sensitization [31].

Here, we used resting-state fMRI to explore alterations in the brain regions, and similar results were obtained by different methods. First, our study revealed lower GMV in patients with AM in the bilateral insula, left angular gyrus, precuneus, left inferior temporal gyrus, and left postcentral gyrus. Further, decreased VMHC was observed in the bilateral insula and postcentral gyrus in the patient group. As a cortical convergence region, the insula participates in various processes, from somatic perception to emotional conflict, receiving information directly from the spinothalamic tract [32,33]. Neurological research suggests that the insular cortex participates in somatosensory and pain processing. Therefore, the reducing GMV of the bilateral insula in patients with pain-related AM may imply the dysfunction of the insula in interoceptive awareness [34]. Furthermore, the angular gyrus, an important region of the default mode network (DMN), is strongly linked to cognition and emotion, especially depression and anxiety [35]. The previous studies demonstrated that the alteration of the DMN might be related to associated symptoms in patients with chronic pain, such as depression, anxiety, and sleep disorders [36]. This study showed that the SDS scores in patients with AM had a significant negative correlation with the GMV of the left angular gyrus, which suggests that the increased degree of depression in patients with pain-related AM could lead to a lower angular-gyrus volume.

Based on the resting-state fMRI, we analyzed the different functions between the patients with pain-related AM and the pain-free controls in VMHC and ReHo. The medial prefrontal cortex (MPFC) is known as a key region of pain processing, which is also an important component of the DMN. Here, the decreased ReHo values in MPFC were found in the pain group, consistent with previous findings in patients with chronic pain [37]. A study focusing on neurotransmitters in patients with chronic pain found a decrease in γ-aminobutyric acid and glutamate in the MPFC [38], suggesting that dysregulation of the MPFC is associated with the development and maintenance of pain. Although not statistically significant, there was a negative correlation tendency between SAS, SDS scores, and functional activity of this brain region. We could speculate that the more severe symptoms of anxiety and depression in patients with pain-related AM may be related to the inactivation of the MPFC.

The studies have demonstrated structural and functional abnormalities of the cingulate gyrus in human and rat models of pelvic pain caused by primary dysmenorrhea [39,40]. Using VHMC and ReHo, consistent hypo-activity was found in the cingulate gyrus in the patients with pain-related AM, denoting that it is important in regulating sensory afferents and pain processing. Further, decreased ReHo values in the bilateral cerebellum was found in patients with pain compared to pain-free participants. The cerebellum is an intermediate of the pain information transduction pathway, transmits pain information to the cerebral cortex through fibrous connections, and participates in the regulation of pain inhibitory pathways [41]. Our results suggest that patients with pain-related AM exhibited changes in multiple brain regions associated with pain as well as emotion perception and processing. Although endometriosis was excluded from the inclusion criteria, the coexistence of endometriosis and AM is relatively frequent. We will explore the brain changes for this condition in the future and compare it with this study, which might provide new insights into the relationship between AM and endometriosis.

Our study had some limitations. First, the sample size was not sufficiently large, and we did not perform a power analysis; more convincing results may be obtained by increasing the number of participants. Second, more studies are needed to verify the reliability and repeatability of this study. Thirdly, in the control group, adenomyosis was excluded by gynecologic ultrasound but not pathology. Finally, longitudinal studies are needed to investigate whether the changes in the brain regions are caused by persistent pain or changes leading to hyperalgesia in patients with AM.

## 5. Conclusions

We preliminarily demonstrated that pain leads to morphological and functional changes in brain regions related to pain in patients with AM. This could offer novel insight into understanding the neural mechanism of pain in patients with AM and be helpful for the objective assessment and individualized treatment of patients with pain-related AM.

## Figures and Tables

**Table 1:** Demographic and psychophysical characteristics of the participants.

Characteristic	Adenomyosis (*n* = 49)	Controls (*n* = 30)	*p*-Value
Age (y)	40.52 ± 6.28	39.27 ± 7.33	0.420
BMI (kg/m^2^)	24.67 ± 3.69	24.77 ± 3.43	0.906
Age of menarche (y)	13.40 ± 1.43	13.27 ± 1.20	0.914
Menstrual cycle (d)	29.88 ± 8.85	28.80 ± 4.06	0.534
Menstruation (d)	6.35 ± 2.33	5.87 ± 1.40	0.508
Menorrhagia *n* (%)	26 (52.00%)	4 (13.33%)	0.001 *
Gravidity	2.98 ± 1.70	3.27 ± 1.72	0.437
Uterine volume (cm^3^)	327.19 ± 147.05	98.59 ± 45.32	<0.001 *
HGB (g/L)	104.53 ± 20.07	122.27 ± 16.59	<0.001 *
NRS	8.40 ± 1.78	0.43 ± 0.82	<0.001 *
SAS	33.38 ± 8.17	29.16 ± 6.29	0.018 *
SDS	35.96 ± 9.80	31.13 ± 8.32	0.027 *

* Significant differences (*p*) determined using an unpaired *t*-test for normally distributed data, and otherwise with the Mann–Whitney U test. BMI, body mass index; HGB, hemoglobin; NRS, numeric rating scale; SAS, self-rating anxiety scale; SDS, self-rating depression scale.

## Data Availability

The data presented in this study are available on request from the corresponding authors.

## Supplementary Materials

The following supporting information can be downloaded at: https://www.mdpi.com/article/10.3390/jcm11185286/s1, File S1: Imaging data acquisition and processing. Reference [42] cited in Supplementary Materials.
