# Supplementary material for: Brain Morphological and Functional Changes in Adenomyosis with Pain: A Resting State Functional Magnetic Resonance Imaging Study
Source: J Clin Med. 2022 Sep 7;11(18):5286. doi: 10.3390/jcm11185286 (PMC9504732; doi:10.3390/jcm11185286)
Supplement: Supplementary file 1 [file jcm-11-05286-s001.zip › jcm-1881080-supplementary.pdf]

Imaging data acquisition and processing

Resting-state fMRI data were obtained using Gradient-Echo Single-Shot Echo-Planar Imaging sequence (GRE-SS-EPI) with the determined imaging parameters: repetition time (TR)/echo time (TE) = 2000/30 ms; field of view (FOV) = 220 mm × 220 mm; matrix = 64 × 64; flip angle (FA) = 90°; slice thickness = 3 mm; and slice gap = 1mm; 36 transversal slices; 180 volumes. Sagittal 3D T1-weighted images were acquired by magnetization prepared rapid acquisition gradient echo (MPRAGE) sequence: TR/TE = 2000/2.3 ms; inversion time = 900 ms; FA = 9°; matrix = 256 × 256; slice thickness = 1 mm, no gap; 192 slices.

To reach the signal equilibrium, the first 10 volumes of each functional time series were discarded. The remaining 170 volumes were corrected for the acquisition time delay between different slices and further realigned to the first volume. Head movement parameters were acquired through estimating the translation in each direction and the angular rotation on each axis for each volume. Overall, one individuals were excluded from further analysis due to a maximum displacement in any orthogonal directions greater than 3 mm or a maximum head rotation greater than 3.0°. As described in previous study, small amounts of head motion from volume to volume could have an impact on the FC results [42]. Framewise displacement (FD) was also calculated, indexing volume-to-volume displacement in head position. The FD was calculated from the derivatives of the rigid body realignment estimates which were used to realign fMRI data [42]. We carefully checked the FD of every individuals and one participant with an FD greater than 0.5 was then excluded from further analyses. Then, the individual high-resolution structural images were coregistered to the mean resting-state fMRI image after motion correction with a linear trans- formation. The transformed structural images were then segmented into gray matter, white matter, and cerebrospinal fluid using a unified segmentation algorithm. In addition, a brain template was

generated through diffeomorphic anatomical registration through exponential Lie algebra. The movement-corrected functional volumes were spatially normalized to the Montreal Neurological Institute (MNI) space and resampled to 3-mm3 isotropic voxels using the normalization parameters estimated during the unified segmentation. The normalized fMRI data were smoothed with 6-mm full width at half maximum.

Reference

42. Power, J.D.; Barnes, K.A.; Snyder, A.Z.; Schlaggar, B.L.; Petersen, S.E. Spurious but systematic correlations in functional connectivity MRI networks arise from subject motion. *Neuroimage* **2012**, *59*, 2142–2154. http://doi:10.1016/j.neuroimage.2011.10.018.
